# Dietary supplementation with N‐3 polyunsaturated fatty acid‐enriched fish oil promotes wound healing after ultraviolet B‐induced sunburn in mice

**DOI:** 10.1002/fsn3.2330

**Published:** 2021-05-17

**Authors:** Fanxing Meng, Jiayi Qiu, Houjie Chen, Xiaojun Shi, Meifang Yin, Meishu Zhu, Guang Yang

**Affiliations:** ^1^ College of Animal Science and Technology Jilin Agricultural University Changchun China; ^2^ Sciences Po Paris France; ^3^ The Shenzhen Key Laboratory of Health Sciences and Technology International Graduate School at Shenzhen Tsinghua University Shenzhen China; ^4^ Department of Burn and Plastic Surgery Department of Wound Repair Shenzhen Institute of Translational Medicine The First Affiliated Hospital of Shenzhen University Health Science Center Shenzhen Second People's Hospital Shenzhen China

**Keywords:** antioxidant enzymes, inflammation, macrophage polarization, n‐3 polyunsaturated fatty acids, sunburn

## Abstract

N‐3 polyunsaturated fatty acids (n‐3 PUFA) can alleviate ultraviolet B (UVB)‐induced skin cancers, but their effects on sunburn and upcoming wound healing remain controversial. This study aimed to explore the impact of n‐3 PUFA‐enriched fish oil (n‐3 PUFA‐FO) on UVB‐induced sunburns and subsequent healing. Sixty C57BL/6 female mice were divided into two groups. The treated group mice were fed n‐3 PUFA‐FO for the entire duration of the experiment. Mice in the control group were fed a standard diet. After two weeks of n‐3 PUFA‐FO feeding, mice were exposed to UVB for 20 min and sacrificed 20 d later. Skin photodamage and lesion area were recorded during wound healing. Epidermal lesion thickness was quantified in hematoxylin and eosin‐stained skin sections. Inflammation and macrophage polarization were assessed by qRT‐PCR. Oxidative stress and antioxidant enzyme activity were quantified using specific ELISA kits. N‐3 PUFA‐FO feeding decreased UVB photodamage and accelerated wound healing progression, both of which were coupled with less intense inflammation and increased macrophage M2 phenotype polarization. Furthermore, n‐3 PUFA‐FO brought about a decrease in malondialdehyde (MDA) levels but increased the activity of catalase (CAT) and glutathione peroxidase (GP), without changing superoxide dismutase (SOD) activity. N‐3 PUFA‐FO protects against UVB‐induced skin photodamage and promotes wound healing by modulating macrophage phenotypic polarization and antioxidant enzyme activities. N‐3 PUFA‐FO could be a novel therapeutic approach for both the prevention and treatment of sunburns.

## Graphical abstract

Omega‐3 polyunsaturated fatty acids‐enriched fish oil protect from UVB‐induced skin photodamage and advance wound healing by modulating macrophages phenotypic polarization and antioxidant enzymes activities. It could embody a novel therapeutic approach to both the prevention and the treatment of sunburns.

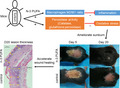

## 1 INTRODUCTION

Sunburns represent a significant public health problem because they are associated with several types of skin injury and a variety of skin cancers (Lim & Robson, 2001; Narayanan et al., 2010; Zalaudek et al., 2020). Ultraviolet B (UVB) rays, which are the most damaging spectrum of solar irradiation, can severely damage various skin structures, resulting in erythema, edema, hyperplasia, photoaging, and even skin cancers (Bernard et al., 2019). These pathophysiological processes are usually considered to be the consequence of UVB‐induced inflammation, oxidative stress, DNA damage, immunosuppression, and structurally destructive alterations of the skin (Bernard et al., 2019; Moore et al., 2013; Wagener et al., 2013).

The balance between omega‐3 and omega‐6 polyunsaturated fatty acids (n‐3, n‐6 PUFA) is crucial to human health (DeFilippis & Sperling, 2006; García‐Esquinas et al., 2019; Hooper et al., 2006). Eicosapentaenoic acid (EPA) and docosahexaenoic acid (DHA) derived from fish oil are the two main types of n‐3 PUFAs. An increased intake of n‐3 PUFA can reduce the occurrence of dermatitis, melanogenesis, allergies, and skin cancer (Huang et al., 2018; Ilievska et al., 2016; Rodriguez‐Cruz & Serna, 2017). These benefits are closely associated with less intense inflammation and diminished oxidative stress (de Bus et al., 2019). Therefore, n‐3 PUFAs have been widely used as nutritional supplements and as ingredients in skincare products.

The long lifespan of humans has led to changes in eating habits that alter the balance between n‐3 PUFA and n‐6 PUFA. Modern diets are devoid of n‐3 PUFAs. Moreover, mammals are unable to synthesize PUFAs (Kang, 2003). These realities increase the risks involved in exposing the skin to sunlight. Although n‐3 PUFA can reduce UVB‐induced skin diseases, the role of n‐3 PUFAs in sunburn and subsequent wound healing remains controversial (McDaniel et al., 2008). Therefore, this study aims to clarify whether an increased intake of n‐3 PUFA‐FO can safeguard skin health following UVB irradiation injury.

Herein, we report the results of investigating the effects of dietary supplementation with n‐3 PUFA‐FO on UVB‐induced acute skin damage and the subsequent wound healing progression, skin structure alterations, oxidative stress, and inflammation.

## 2 MATERIALS AND METHODS

### 2.1 Ethical statements and animals

All animal experiments were approved by the Institutional Animal Care and Use Committee of Jilin Agricultural University. Protocols were approved by all relevant guidelines and laws. The mice were anesthetized with isoflurane (970‐00026‐00, RWD, Shenzhen, China) and euthanized using CO_2_. Animals were housed individually in a temperature‐controlled room at 24 ± 2°C, with 40%–60% humidity and a 12 hr light/dark cycle, and fed ad libitum.

**FIGURE 1:**
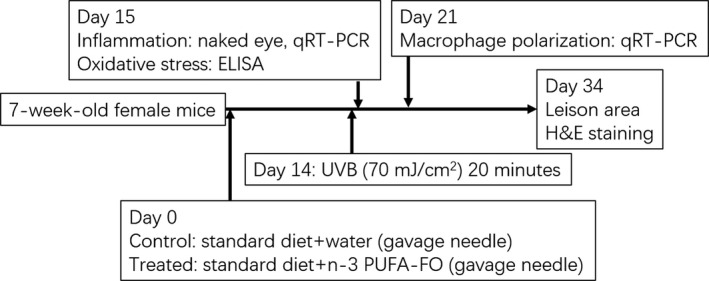
Experimental flowchart

Sixty seven‐week‐old C57BL/6 littermate female mice were divided into the treated and control groups. The treated group mice received a normal diet (1,025, HFK, Beijing, China) supplemented with n‐3 PUFA‐FO (100 μl/day; 18% EPA and 12% DHA, Piping Rock, NY, USA). Mice in the control group were solely fed a standard diet. The treated group was administered n‐3 PUFA‐FO (50 μl/day) by gavage needle, and the control group was given the same amount of water by gavage needle. After 2 weeks of staying in the animal house, both mice groups were exposed to UVB (70 mJ/cm^2^) for 20 min. The treated mice were fed n‐3 PUFA‐FO for the entire duration of the experiments. The skin tissues were collected for subsequent studies as required. The experimental steps are shown in Figure 1.

### 2.2 Skin lesions

Mice were anesthetized for observation on days 5 (D5), 10 (D10), 15 (D15), and 20 (D20) post‐irradiation. Images were taken with a cell phone (Mate 9, Huawei, Shenzhen, China) and analyzed using ImageJ software (Version 1.52; NIH, USA).

### 2.3 Histology

At termination, the mouse dorsal skin was sampled and preserved in 4% paraformaldehyde (PFA) overnight. Tissue sections (8 μm thick) from paraffin (40,631,172, Citotest, Jiangsu, China)‐embedded skin samples were stained with hematoxylin and eosin (H&E) (PH1732, Phygene, Fuzhou, China). The slides were observed under a Zeiss microscope (Axiocam 503, Zeiss, Jena, Germany) and images were captured. The images were analyzed using Zen lite software (version 2.1, Zeiss, Jena, Germany).

### 2.4 Quantitative real time‐PCR (qRT‐PCR)

Skin samples were collected and preserved in RNA‐wait (BL621A, Biosharp, Hefei, China) at 4℃ overnight. The next day, samples were transferred to −80 ℃ for long‐term storage until the next step. Total RNA was isolated from mouse dorsal skin samples using TRIzol^®^ reagent (9,109, Takara, Kusatsu, Japan). After extraction, RNA was quantified using a Nanodrop^TM^ Spectrophotometer (NanoDrop One, Thermo Scientific, WI, USA). The cDNA was synthesized using the PrimeScript RT reagent Kit (RR047A, Takara, Kusatsu, Japan). The qRT‐PCR was performed in an ABI‐7300 (ABI, Foster City, CA, USA) using SYBR Green (B21203, Bimake, Shanghai, China) according to the manufacturer's instructions. Briefly, the final reaction volume was 20 μL (10 μL Master Mix, 1 μL forward primer, 1 μL reverse primer, 100 ng cDNA, 0.4 μL ROX Reference Dye, deionized water) for each gene under the following thermal conditions: 95°C for 10 min, 95°C for 15 s, 60°C for 60 s) for 40 cycles. Samples without reverse transcription were used as the negative controls. *Gapdh* was used as the control housekeeping gene. The primer sequences used are shown in Table 1.

**TABLE 1:** List of primers used for qRT‐PCR

Gene name	Forward (from 5' to 3')	Reverse (from 5' to 3')
*Il‐1b*	5'‐AAGAGCTTCAGGCAGGCAGTATCA‐3'	5'‐TGCAGCTGTCTAATGGGAACGTCA‐3'
*Il‐6*	5'‐TCTATACCACTTCACAAGTCGGA‐3'	5'‐GAATTGCCATTGCACAACTCTTTC‐3'
*Mcp1*	5'‐CCAGCCTACTCATTGGGATCA‐3'	5'‐CTTCTGGGCCTGCTGTTCA‐3'
*Tnf‐a*	5'‐ACGTCGTAGCAAACCACCAA‐3'	5'‐GCAGCCTTGTCCCTTGAAGA‐3'
*Ym1*	5'‐ACTTTGATGGCCTCAACCTGGACT‐3'	5'‐TGGAAGTGAGTAGCAGCCTTGGAA‐3'
*Fizz1*	5'‐ACTGCCTGTGCTTACTCGTTGACT‐3'	5'‐AAAGCTGGGTTCTCCACCTCTTCA‐3'
*Arg1*	5'‐ACCTGGCCTTTGTTGATGTCCCTA‐3'	5'‐AGAGATGCTTCCAACTGCCAGACT‐3'
*Mgl2*	5'‐TTAGCCAATGTGCTTAGCTGG‐3'	5'‐GGCCTCCAATTCTTGAAACCT‐3'
*Mrc1*	5'‐GTGCTGGTTGTGATAGCCATC‐3'	5'‐TGCTGACACTTACCATCAGGT‐3'
*Slamf1*	5'‐CAGAAATCAGGGCCTCAAGAG‐3'	5'‐CACTGGCATAAACTGTGGTGG‐3'
*Il12p40*	5'‐TGGTTTGCCATCGTTTTGCTG‐3'	5'‐ACAGGTGAGGTTCACTGTTTCT‐3'
*Il‐1r*	5'‐GTGCTACTGGGGCTCATTTGT‐3'	5'‐GGAGTAAGAGGACACTTGCGAAT‐3'
*NF‐κB*	5'‐TCCACTGTCTGCCTCTCTCGTC‐3'	5'‐GCCTTCAATAGGTCCTTCCTGC‐3'
*Ccl5*	5'‐GTGCTCCAATCTTGCAGTCG‐3'	5'‐AGAGCAAGCAATGACAGGGA‐3'
*Gapdh*	5'‐AGGTCGGTGTGAACGGATTTG‐3'	5'‐GGGGTCGTTGATGGCAACA‐3'

### 2.5 ELISA

Skin samples were collected, weighed, and then homogenized by ultrasound treatment (JY92‐IIN, Ningbo Scientz, Ningbo, China) in cold Tris‐HCl (5 mmol/L, containing 2 mmol/L EDTA, pH 7.4). Homogenates were centrifuged at 3,000 rpm for 15 min at 4°C, and the supernatants were stored at −80°C. Protein content was assessed using a BCA protein assay kit (P0012, Beyotime, Beijing, China). The superoxide dismutase (SOD), catalase (CAT), and glutathione peroxidase (GP) enzymatic activity were quantified; and malondialdehyde (MDA) levels were measured using enzyme‐linked immunosorbent assay (ELISA) kits (A001, A007, A005, A003‐1, Nanjing Jiancheng Bioengineering Institute, Nanjing, China). Optical density (OD) values were measured using a microplate reader (Epoch, BioTek, Winooski, Vermont, USA).

### 2.6 Statistical analysis

Data are means ±standard deviations (SDs). Using GraphPad Prism software (Version 6, San Diego, CA, USA), an unpaired two‐tailed Student's *t*‐test was used for statistical analyses. Differences were considered statistically significant at *p* ≤ .05.

## 3 RESULTS

### 3.1 N‐3 PUFA‐FO mitigated UVB‐induced sunburns and accelerated wound healing

**FIGURE 2:**
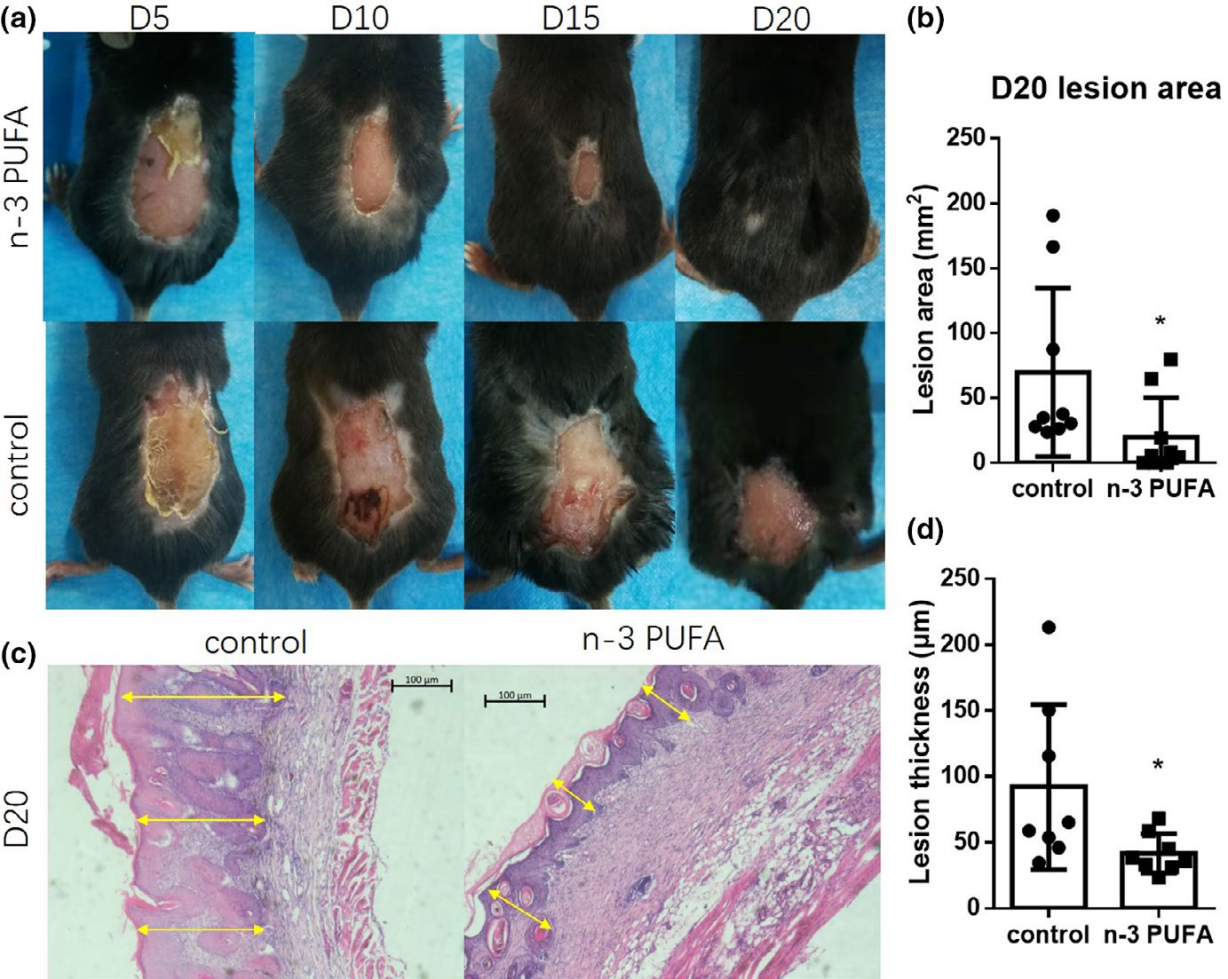
N‐3 PUFA‐FO ameliorates UVB‐induced sunburns. (a) Representative photos of post‐sunburn wound healing at day 5 (D5), day 10 (D10), day 15 (D15), and day 20 (D20). (b) N‐3 PUFA‐FO accelerated post‐sunburn wound healing. (mm2; control, 69.61 ± 65.03 vs. *n*‐3 PUFA, 20.18 ± 30.32, *n* = 9) (c) Representative H&E staining photos of skin at D20. Yellow arrows indicate the lesion thickness. (d) N‐3 PUFA‐FO decreased lesion thickness. (μm; control, 92.25 ± 62.5 vs. n‐3 PUFA, 41.79 ± 14.87, *n* = 8) * *p* ≤ .05

To determine the effects of n‐3 PUFA‐FO in sunburns, we kept the mice for 20 days after UVB exposure. The results showed that n‐3 PUFA‐FO significantly reduced the damage intensity and accelerated wound healing (*p* ≤ .05, Figure 2a,b). To confirm the results further, D20 UVB‐exposed skin samples were collected, and their histological sections were stained with H&E. N‐3 PUFA‐FO significantly decreased the thickness of the damaged skin, indicating that n‐3 PUFA‐FO ameliorated UVB‐induced sunburns and accelerated wound healing (*p* ≤ .05, Figure 2c,d).

### 3.2 N‐3 PUFA‐FO attenuated UVB‐induced inflammation

**FIGURE 3:**
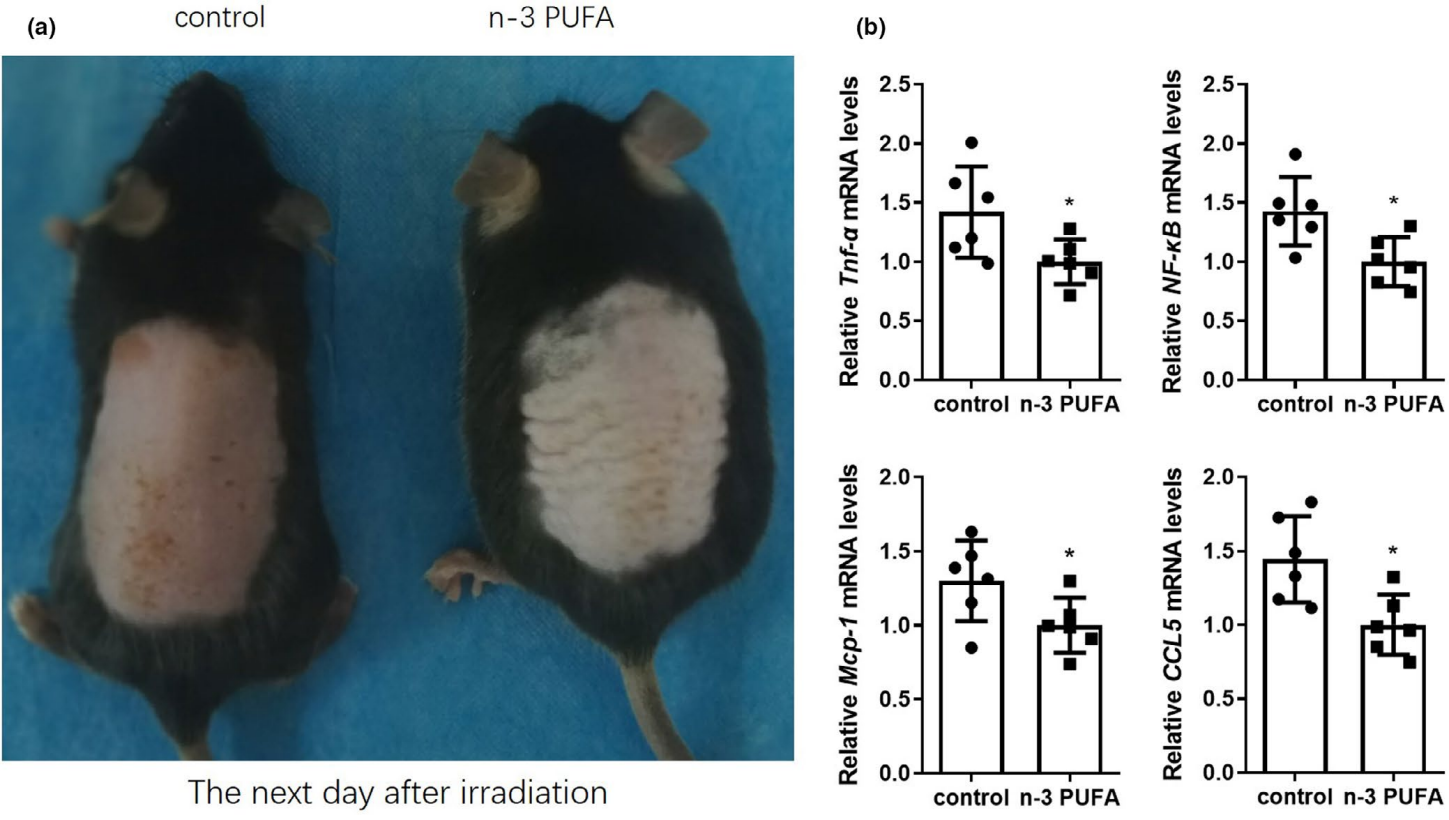
N‐3 PUFA‐FO attenuated the intensity of UVB‐induced inflammation. (a) Representative images of the dorsal skin of the mice, 24 h after sunburn. (b) N‐3 PUFA‐FO decreased *Tnf‐a* (control, 1.42 ± 0.3826 vs. n‐3 PUFA, 1 ± 0.1895, *n* = 6), *NF‐kB* (control, 1.428 ± 0.2896 vs. n‐3 PUFA, 1 ± 0.207 5, *n* = 6), *Mcp‐1* (control, 1.3 ± 0.2726 vs. n‐3 PUFA, 1 ± 0.1852, *n* = 6), and *Ccl5* (control, 1.443 ± 0.2921 vs. *n*‐3 PUFA, 1 ± 0.2039, *n* = 6) genes expression levels 24 h after sunburn. *p ≤ .05

Sunburn is associated with inflammation, and n‐3 PUFAs can reduce inflammation. To investigate whether n‐3 PUFA‐FO helped with the healing of sunburns by decreasing inflammation intensity, skin samples were collected and measured. Twenty‐four hours after UVB exposure, skin erythema was visible to the naked eye. Clearly, n‐3 PUFA‐FO decreased the intensity of UVB‐induced erythema (Figure 3a). To confirm these results, skin samples were collected and analyzed using qRT‐PCR. The results showed that n‐3 PUFA significantly decreased the expression levels of genes encoding various pro‐inflammatory cytokines (*Tnf‐a, NF‐kB*) and chemokines (*Mcp‐1, Ccl5*) (*p* ≤ .05, Figure 3b).

### 3.3 N‐3 PUFA‐FO shifted macrophage phenotype toward M2 during wound healing

**FIGURE 4:**
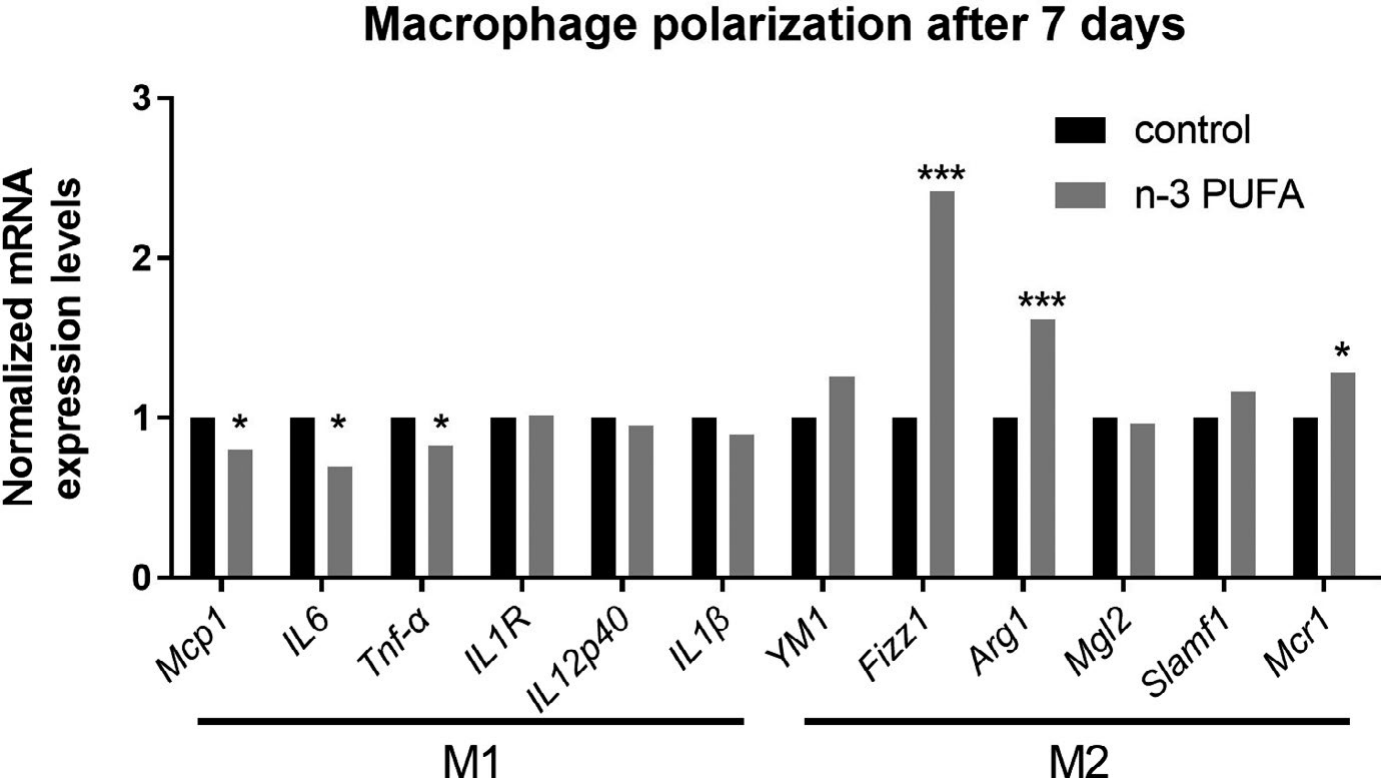
N‐3 PUFA‐FO induced macrophages to shift toward the M2 phenotype. (a) Expression levels of M1 and M2 biomarkers 7 days post‐sunburn. (*n* = 6) * *p* ≤ .05; **p ≤ .01; ***p ≤ .005

There are two main subtypes of macrophages, M1 and M2. M1 macrophages are mainly involved in pro‐inflammatory responses in the early stages of wound healing, and M2 macrophages are mainly involved in anti‐inflammatory responses in the later stages of wound healing. Previous studies have shown that n‐3 PUFA enhances M2 macrophage polarization (Song et al., 2016), which contributes to tissue repair. To confirm that n‐3 PUFA‐FO can push the macrophages to the M2 phenotype after sunburn, M1 and M2 biomarkers were measured by qRT‐PCR. Seven days after the sunburn, the n‐3 PUFA‐FO‐treated group showed a lower expression of M1 markers and a higher expression of M2 markers (Figure 4).

### 3.4 N‐3 PUFA‐FO attenuated UVB‐induced oxidative stress

**FIGURE 5:**
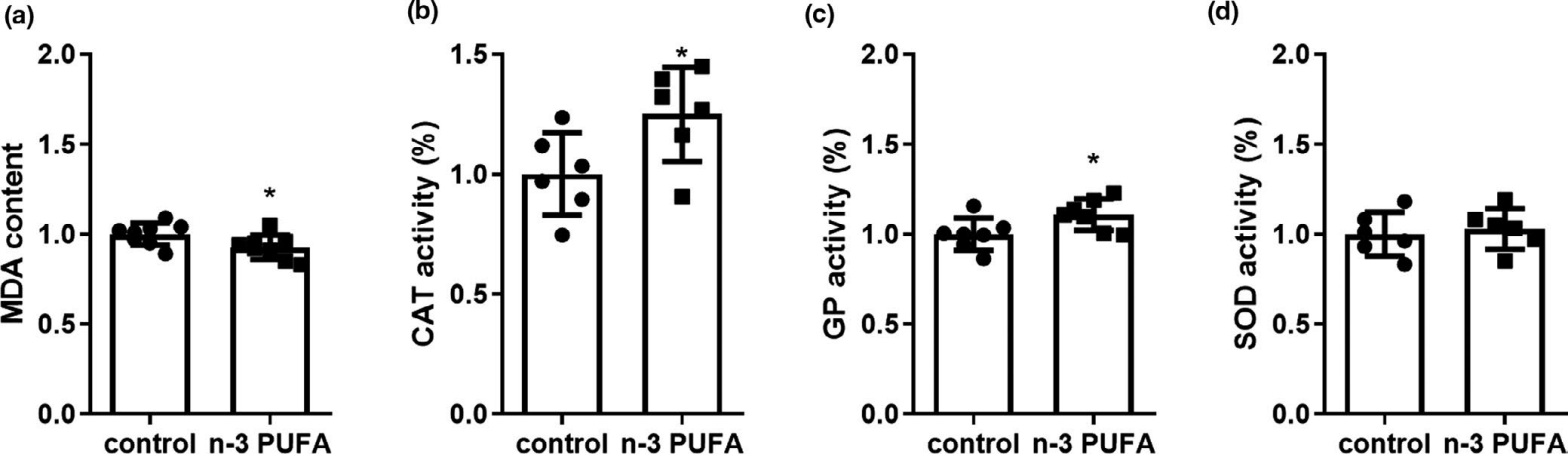
N‐3 PUFA decreased UVB‐induced oxidative stress. (a) Levels of malondialdehyde (MDA). (control, 1 ± 0.06164 vs. *n*‐3 PUFA, 0.9263 ± 0.06823, n = 6–8) (b, c, d) Activity of catalase (CAT) (control, 1 ± 0.1718 vs. *n*‐3 PUFA, 1.25 ± 0.1964, *n* = 6–8), glutathione peroxidase (GP) (control, 1 ± 0.08942 vs. *n*‐3 PUFA, 1.108 ± 0.08683, n = 6–8), superoxide dismutase (SOD) (control, 1 ± 0.1221 vs. *n*‐3 PUFA, 1.03 ± 0.1138, *n *= 6–8). * *p* ≤ .05

Accumulation of macrophages promotes the production and release of ROS (Castaneda et al., 2017), which are harmful to the skin (Rinnerthaler et al., 2015). To investigate the role of n‐3 PUFA‐FO in UVB‐induced oxidative stress, we measured the biomarker MDA and assessed the activity of related antioxidant enzymes 24 hr after sunburn. The results showed that n‐3 PUFA‐FO consumption markedly decreased MDA levels (*p* ≤ .05, Figure 5a) while increasing the activity of the antioxidant enzymes CAT and GP (*p* ≤ .05, Figure 5b,c); however, it did not change SOD activity (Figure 5d).

## 4 DISCUSSION

Although sunburns entail acute photodamage, they are often difficult to perceive before tissue injury occurs. People usually prefer to use physical sunscreens to avoid sunburns, and medications to repair any subsequent photodamage. However, the risk of skin photodamage remains high. This study shows that n‐3 PUFA‐FO can mitigate sunburns by attenuating inflammation intensity and by increasing the activities of antioxidant enzymes CAT and GP. In addition, they demonstrated that n‐3 PUFA‐FO can accelerate sunburn wound healing by pushing macrophages to shift toward the M2 phenotype. Our results justify the development of new sunscreen and skin repair products. Consumption of fish oil supplements can increase skin protection, especially in the case of people who are often exposed to the sun and its UVB rays.

It is well known that macrophages are one of the main producers of ROS and that increased oxidative stress accelerates skin damage and skin aging. Previous studies have shown that n‐3 PUFAs not only reduce inflammatory factors and oxidative stress, but also increase the expression of antioxidant proteins, such as Nrf2, heme oxygenase‐1, NAD(P)H:quinone oxidoreductase‐1, and thioredoxin‐1 (Naoya et al., 2010; You‐Rong et al., 2011; Yum et al., 2017, 2018). In line with this, our study shows that the reduction of inflammation intensity occurred with a simultaneous decrease in the levels of MDA, a marker of oxidative stress. Moreover, n‐3 PUFA‐FO increased the activity of antioxidant enzymes such as CAT and GP at the same time. Comparable results were observed in other tissues, but to the best of our knowledge, this is the first time that such mechanisms have been shown to be functionally observed in the skin. Thus, we posit that the protective role of n‐3 PUFA is based on a reduction in pro‐inflammatory cytokines and oxidative stress, and increased activity of antioxidant enzymes.

In addition to revealing an anti‐sunburn action, our results also highlighted the long‐term effect of n‐3 PUFA‐FO on post‐sunburn wound healing. Several studies have shown that n‐3 PUFA promotes wound healing and is associated with a decreased macrophage M1 phenotype and increased M2 phenotype, fibroblast distribution, and collagen fiber (De Boer et al., 2015; Hankenson et al., 2000; Jacobi et al., 2012; Peng et al., 2018; Song et al., 2016). Generally, macrophage subsets are involved in a variety of functions (Hamilton et al., 2014). M1 macrophages accumulate in the early stages of injury and drive the inflammatory response. Later on, the proportion of M2 macrophages will gradually increase, and by mitigating inflammation, advance tissue repair (Benoit et al., 2008). Our results clearly showed that the n‐3 PUFA‐FO group had a weaker inflammatory reaction on the first day after sunburn, which prompted us to assess the two macrophage phenotypes at D7 post‐sunburn. These results are consistent with our hypothesis regarding the benefits of n‐3 PUFA‐FO.

However, some studies put forward a different view, that is that n‐3 PUFA can inhibit wound repair and increase inflammation intensity (McDaniel et al., 2008). This discrepancy may be related to differences in the models, study methods, and injury types. Mechanical injury causes loss of skin tissue. This type of wound healing involves cell dedifferentiation, transdifferentiation, and differentiation. Tissue regeneration is related to stem cells. Age, regenerative microenvironment, and oxidative stress may affect the growth, migration, and differentiation of stem cells. The healing mechanism of sunburn differs from that of mechanical injury; hence, the results are dissimilar. However, our results collectively confirm the beneficial effects of n‐3 PUFAs on sunburns because of their influence on macrophage phenotypes and oxidative stress.

Furthermore, ROS and macrophages can modulate each other (Tan et al., 2016). ROS crucially regulates monocyte recruitment and macrophage polarization, while macrophage subsets notably affect the generation and fate of ROS. Therefore, we assume that the observed decreases in the inflammatory response and oxidative stress, and the enhanced activity of the two antioxidant enzymes reduced the accumulation of M1 macrophages by curtailing ROS production.

## 5 CONCLUSIONS

In conclusion, our results show that n‐3 PUFA‐FO protects against UVB‐induced photodamage and promotes sunburn wound healing by modulating macrophage phenotypic polarization and antioxidant enzyme activity. Therefore, n‐3 PUFA‐FO can be used as a nutritional supplement and as a prophylactic agent to maintain skin health and homeostasis.

## CONFLICT OF INTEREST

The authors do not have any conflicts of interest to declare.

## AUTHOR CONTRIBUTION

**Fanxing Meng:** Data curation (equal); Investigation (equal); Methodology (equal). **Jiayi Qiu:** Formal analysis (equal); Investigation (equal). **Houjie Chen:** Methodology (equal). **Xiaojun Shi:** Methodology (equal). **Meifang Yin:** Writing‐review & editing (equal). **Meishu Zhu:** Writing‐review & editing (equal). **​Guang ​Yang:** Funding acquisition (equal); Methodology (equal); Supervision (equal); Writing‐original draft (equal); Writing‐review & editing (equal).

## Data Availability

The data that support the findings of this study are available from the corresponding author upon reasonable request.

## References

[fsn32330-bib-0001] Benoit, M. , Desnues, B. , & Mege, J. L. (2008). Macrophage polarization in bacterial infections. The Journal of Immunology, 181(6), 3733–3739. 10.4049/jimmunol.181.6.3733 18768823

[fsn32330-bib-0002] Bernard, J. J. , Gallo, R. L. , & Krutmann, J. (2019). Photoimmunology: How ultraviolet radiation affects the immune system. Nature Reviews Immunology, 19(11), 688–701. 10.1038/s41577-019-0185-9 31213673

[fsn32330-bib-0003] Castaneda, O. A. , Lee, S. C. , Ho, C. T. , & Huang, T. C. (2017). Macrophages in oxidative stress and models to evaluate the antioxidant function of dietary natural compounds. J Food Drug Anal, 25(1), 111–118. 10.1016/j.jfda.2016.11.006 28911528 PMC9333431

[fsn32330-bib-0004] De Boer, A. A. , Monk, J. M. , Liddle, D. M. , Power, K. A. , Ma, D. W. , & Robinson, L. E. (2015). Fish Oil‐Derived Long‐Chain n‐3 Polyunsaturated Fatty Acids Reduce Expression of M1‐Associated Macrophage Markers in an ex vivo Adipose Tissue Culture Model, in Part through Adiponectin. Front Nutr, 2, 31. 10.3389/fnut.2015.00031 26528480 PMC4602148

[fsn32330-bib-0005] de Bus, I. , Witkamp, R. , Zuilhof, H. , Albada, B. , & Balvers, M. (2019). The role of n‐3 PUFA‐derived fatty acid derivatives and their oxygenated metabolites in the modulation of inflammation. Prostaglandins & Other Lipid Mediators, 144, 106351. 10.1016/j.prostaglandins.2019.106351 31260750

[fsn32330-bib-0006] DeFilippis, A. P. , & Sperling, L. S. (2006). Understanding omega‐3's. American Heart Journal, 151(3), 564–570. 10.1016/j.ahj.2005.03.051 16504616

[fsn32330-bib-0007] García‐Esquinas, E. , Ortolá, R. , Banegas, J. R. , Lopez‐García, E. , & Rodríguez‐Artalejo, F. (2019). Dietary n‐3 polyunsaturated fatty acids, fish intake and healthy ageing. International Journal of Epidemiology, 48(6), 1914–1924. 10.1093/ije/dyz196 31563961

[fsn32330-bib-0008] Hamilton, T. A. , Zhao, C. , Pavicic, P. G. Jr , & Datta, S. (2014). Myeloid colony‐stimulating factors as regulators of macrophage polarization. Frontiers in Immunology, 5, 554. 10.3389/fimmu.2014.00554 25484881 PMC4240161

[fsn32330-bib-0009] Hankenson, K. D. , Watkins, B. A. , Schoenlein, I. A. , Allen, K. G. , & Turek, J. J. (2000). Omega‐3 fatty acids enhance ligament fibroblast collagen formation in association with changes in interleukin‐6 production. Proceedings of the Society for Experimental Biology and Medicine, 223(1), 88–95. 10.1046/j.1525-1373.2000.22312.x 10632966

[fsn32330-bib-0010] Hooper, L. , Thompson, R. L. , Harrison, R. A. , Summerbell, C. D. , Ness, A. R. , Moore, H. J. , Worthington, H. V. , Durrington, P. N. , Higgins, J. P. T. , Capps, N. E. , Riemersma, R. A. , Ebrahim, S. B. J. , & Smith, G. D. (2006). Risks and benefits of omega 3 fats for mortality, cardiovascular disease, and cancer: Systematic review. BMJ, 332(7544), 752–760. 10.1136/bmj.38755.366331.2F 16565093 PMC1420708

[fsn32330-bib-0011] Huang, T. H. , Wang, P. W. , Yang, S. C. , Chou, W. L. , & Fang, J. Y. (2018). Cosmetic and Therapeutic Applications of Fish Oil's Fatty Acids on the Skin. Mar Drugs, 16(8), 10.3390/md16080256 PMC6117694 30061538

[fsn32330-bib-0012] Ilievska, B. , Loftsson, T. , Hjalmarsdottir, M. A. , & Asgrimsdottir, G. M. (2016). Topical Formulation Comprising Fatty Acid Extract from Cod Liver Oil: Development, Evaluation and Stability Studies. Mar Drugs, 14(6), 10.3390/md14060105 PMC4926064 27258290

[fsn32330-bib-0013] Jacobi, S. K. , Moeser, A. J. , Corl, B. A. , Harrell, R. J. , Blikslager, A. T. , & Odle, J. (2012). Dietary long‐chain PUFA enhance acute repair of ischemia‐injured intestine of suckling pigs. Journal of Nutrition, 142(7), 1266–1271. 10.3945/jn.111.150995 PMC7289324 22623387

[fsn32330-bib-0014] Kang, J. X. (2003). The importance of omega‐6/omega‐3 fatty acid ratio in cell function. The gene transfer of omega‐3 fatty acid desaturase. World Review of Nutrition and Dietetics, 92, 23–36. 14579681 10.1159/000073790

[fsn32330-bib-0015] Lim, H. W. , & Robson, K. J. (2001). Acute and Chronic Photodamage from Solar Radiation, Phototherapy. and Photochemotherapy.

[fsn32330-bib-0016] Lou, Y.‐R. , Peng, Q.‐Y. , Li, T. , Medvecky, C. M. , Lin, Y. , Shih, W. J. , Conney, A. H. , Shapses, S. , Wagner, G. C. , & Lu, Y.‐P. (2011). Effects of high‐fat diets rich in either omega‐3 or omega‐6 fatty acids on UVB‐induced skin carcinogenesis in SKH‐1 mice. Carcinogenesis, 32(7), 1078–1084. 10.1093/carcin/bgr074 21525235 PMC3128560

[fsn32330-bib-0017] McDaniel, J. C. , Belury, M. , Ahijevych, K. , & Blakely, W. (2008). Omega‐3 fatty acids effect on wound healing. Wound Repair Regen, 16(3), 337–345. 10.1111/j.1524-475X.2008.00388.x 18471252 PMC2967211

[fsn32330-bib-0018] Moore, C. , Cevikbas, F. , Pasolli, H. A. , Chen, Y. , Kong, W. , Kempkes, C. , Parekh, P. , Lee, S. H. , Kontchou, N.‐A. , Yeh, I. , Jokerst, N. M. , Fuchs, E. , Steinhoff, M. , & Liedtke, W. B. (2013). UVB radiation generates sunburn pain and affects skin by activating epidermal TRPV4 ion channels and triggering endothelin‐1 signaling. Proc Natl Acad Sci U S A, 110(34), E3225–3234. 10.1073/pnas.1312933110 23929777 PMC3752269

[fsn32330-bib-0019] Naoya, T. , Kazuhiko, T. , Hiroshi, T. , Yuka, I. , Atsushi, I. , Yoichi, F. , & Harumi, O. (2010). Dietary, but not topical, alpha‐linolenic acid suppresses UVB‐induced skin injury in hairless mice when compared with linoleic acids. Photochemistry & Photobiology, 76(6), 657–663. 10.1562/0031-8655(2002)076<0657:dbntal>2.0.co;2 12511046

[fsn32330-bib-0020] Narayanan, D. L. , Saladi, R. N. , & Fox, J. L. (2010). Ultraviolet radiation and skin cancer. International Journal of Dermatology, 49(9), 978–986. 10.1111/j.1365-4632.2010.04474.x 20883261

[fsn32330-bib-0021] Peng, Y. C. , Yang, F. L. , Subeq, Y. M. , Tien, C. C. , Chao, Y. C. , & Lee, R. P. (2018). Lipid Emulsion Enriched in Omega‐3 PUFA Accelerates Wound Healing: A Placebo‐Controlled Animal Study. World Journal of Surgery, 42(6), 1714–1720. 10.1007/s00268-017-4404-x 29264725

[fsn32330-bib-0022] Rinnerthaler, M. , Bischof, J. , Streubel, M. K. , Trost, A. , & Richter, K. (2015). Oxidative stress in aging human skin. Biomolecules, 5(2), 545–589. 10.3390/biom5020545 25906193 PMC4496685

[fsn32330-bib-0023] Rodriguez‐Cruz, M. , & Serna, D. S. (2017). Nutrigenomics of omega‐3 fatty acids: Regulators of the master transcription factors. Nutrition, 41, 90–96. 10.1016/j.nut.2017.04.012 28760435

[fsn32330-bib-0024] Song, M. Y. , Wang, J. , Lee, Y. , Lee, J. , Kwon, K. S. , Bae, E. J. , & Park, B. H. (2016). Enhanced M2 macrophage polarization in high n‐3 polyunsaturated fatty acid transgenic mice fed a high‐fat diet. Molecular Nutrition & Food Research, 60(11), 2481–2492. 10.1002/mnfr.201600014 27306613

[fsn32330-bib-0025] Tan, H. Y. , Wang, N. , Li, S. , Hong, M. , Wang, X. , & Feng, Y. (2016). The Reactive Oxygen Species in Macrophage Polarization: Reflecting Its Dual Role in Progression and Treatment of Human Diseases. Oxid Med Cell Longev, 2016, 2795090. 10.1155/2016/2795090 27143992 PMC4837277

[fsn32330-bib-0026] Wagener, F. A. , Carels, C. E. , & Lundvig, D. M. (2013). Targeting the redox balance in inflammatory skin conditions. International Journal of Molecular Sciences, 14(5), 9126–9167. 10.3390/ijms14059126 23624605 PMC3676777

[fsn32330-bib-0027] Yum, H. W. , Kim, S. H. , Kang, J. X. , & Surh, Y. J. (2018). Amelioration of UVB‐induced oxidative stress and inflammation in fat‐1 transgenic mouse skin. Biochemical and Biophysical Research Communications, 502(1), 1–8. 10.1016/j.bbrc.2018.05.093 29775616

[fsn32330-bib-0028] Yum, H.‐W. , Park, J. , Park, H.‐J. , Shin, J. W. , Cho, Y.‐Y. , Kim, S.‐J. , Kang, J. X. , & Surh, Y.‐J. (2017). Endogenous ω‐3 Fatty Acid Production by fat‐1 Transgene and Topically Applied Docosahexaenoic Acid Protect against UVB‐induced Mouse Skin Carcinogenesis. Scientific Reports, 7(1), 11658. 10.1038/s41598-017-11443-2 28912452 PMC5599646

[fsn32330-bib-0029] Zalaudek, I. , Conforti, C. , Corneli, P. , Jurakic Toncic, R. , di Meo, N. , Pizzichetta, M. A. , Fadel, M. , Mitija, G. , & Curiel‐Lewandrowski, C. (2020). Sun‐protection and sun‐exposure habits among sailors: Results of the 2018 world's largest sailing race Barcolana' skin cancer prevention campaign. Journal of the European Academy of Dermatology and Venereology, 34(2), 412–418. 10.1111/jdv.15908 31442352

